# Testing the integrated motivational–volitional model of suicidal behaviour among Danish adolescents in clinical psychiatric care: focus on volitional moderators

**DOI:** 10.1192/bjo.2026.12066

**Published:** 2026-08-10

**Authors:** Erik Christiansen, Agnieszka Konieczna, Katrine Ulrikke Madsen, Christina P. Larsen, Sarah Grube Jakobsen, Rory C. O’Connor

**Affiliations:** Research Unit of Psychiatry, Department of Regional Health Research, University of Southern Denmark, Odense, Denmark; Centre for Suicide Research, Odense, Denmark; Research Unit of Health Promotion, Department of Public Health, University of Southern Denmark, Esbjerg, Denmark; Suicidal Behaviour Research Laboratory, School of Health and Wellbeing, University of Glasgow, UK

**Keywords:** Self-harm, suicide, child and adolescent psychiatry, structural equation modelling, risk assessment

## Abstract

**Background:**

The integrated motivational–volitional (IMV) model of suicidal behaviour has received growing empirical support, yet few studies have tested its core pathways (defeat → entrapment → suicidal ideation → suicide attempts) in clinical psychiatric adolescent populations. Evidence on volitional moderators influencing the transition from ideation to action remains limited.

**Aims:**

This study aimed to (a) test the core pathways of the IMV model and (b) examine whether volitional moderators differentiate between adolescents with suicidal ideation and those who have attempted suicide.

**Method:**

In this cross-sectional study, 203 Danish adolescents aged 13–19 years were recruited from an out-patient child and adolescent psychiatry department, including patients in treatment or referred for initial assessment. Participants completed validated measures assessing IMV constructs. Path analysis tested the core pathways and multinomial logistic regression examined associations between volitional moderators and group membership (no suicidal behaviour, suicidal ideation only and suicide attempt), with Wald tests comparing effects across outcomes.

**Results:**

Findings supported the core IMV pathway, with the entrapment–ideation pathway being weakest. Volitional moderators were more strongly associated with suicide attempts than suicidal ideation, with significant differences confirmed by Wald tests. A dose–response pattern showed increasing likelihood of suicide attempts with greater exposure to volitional moderators; effect sizes differed significantly between ideation (odds ratio 2.21) and attempts (odds ratio 3.94).

**Conclusions:**

Results support the IMV model and highlight the role of volitional moderators in differentiating between suicidal ideation and attempts. These findings support a shift towards theory-informed psychosocial assessment in clinical adolescent populations.

Suicide represents a significant public health issue, with approximately 727 000 deaths by suicide occurring globally each year. It is the fourth leading cause of death worldwide among young people aged 15–29 years.^1^ In Denmark, suicide is the second leading cause of death among individuals in this age group. For every suicide there are many more suicide attempts, and a suicide attempt is one of the most critical risk factors for future suicide.^2^

Suicidal behaviour often occurs in the context of mental illness. Indeed, a comprehensive review and meta-analysis of 50 years of research on suicide risk factors identified depression and anxiety among the top 5 risk factors for suicidal ideation.^2^ Personality disorders and prior psychiatric hospitalisation were among the top five risk factors for suicide attempts, with prior psychiatric hospitalisation being the foremost risk factor for death by suicide.^2^ These findings highlight robust associations at the group level and underscore the importance of targeting suicide prevention efforts among individuals with mental illness. However, as emphasised by Rose’s distinction between determinants of individual risk and population-level patterns,^3^ such factors may have limited utility for predicting suicidal behaviour at the individual level. Indeed, their ability to accurately identify who will go on to engage in suicidal behaviour is poor and often little better than chance.^2,4^ This limitation underscores the need to move beyond traditional risk factor approaches.

Historically, risk factors for suicidal behaviour have been incorporated into suicide risk assessment instruments to evaluate the risk of future suicidal behaviour and subsequently inform treatment planning. Traditionally these instruments, derived from either standardised scales or statistical modelling, have been used to categorise individuals into three risk levels: low, moderate and high.^5,6^ However, in recent years these instruments have faced substantial criticism for lacking sufficient sensitivity and specificity. Furthermore, the positive predictive value (PPV) – the proportion of those classified as high risk who are truly at high risk – is notably low. One meta-analysis estimated the overall pooled PPV of any instrument to be 16.0% (CI: 12.8–19.8) for any suicidal behaviour combined.^5^ For self-harm (including suicide attempts) it was 26.3% (CI: 21.8–31.3%); for self-harm and suicide combined it was 35.9% (CI: 25.8–47.4%); and for suicide alone it was 5.5% (CI: 3.9–7.9%). When the analysis is restricted to the newest generation of instruments (scales derived from statistical models), the resultant overall PPV is 38.7% (CI: 27–52%). Given that suicide is a rare outcome, it is important to note that the PPV will be inherently low due to its reliance on prevalence.^5^

However, Kessler and colleagues argue, in a critical review of suicide prediction models, that one way to improve prediction accuracy is to gather more information about the risk factors involved and incorporate more comprehensive information to enhance prevention.^7^ Although this may enhance predictive performance, it does not fully address the limitation, highlighted by Rose,^3^ that factors associated with suicidal behaviour at the population level may have limited utility in identifying risk at the individual level. This underscores the need for theoretical models that specify the processes underlying suicidal behaviour to inform treatment planning.

Building on this perspective, theoretical models can help clarify which types of information are most relevant by specifying the psychological processes that lead individuals from suicidal ideation to suicidal behaviour. Over the past 15 years, several ideation-to-action frameworks – including the interpersonal theory of suicide and the three-step theory – have been developed to address this distinction by conceptualising the emergence of ideation and progression to behaviour as separate processes.^8,9^ The integrated motivational–volitional (IMV) model (Fig. 1) extends this work by identifying a set of volitional moderators that are proposed to differentiate those who think about suicide from those who act on these thoughts.^10^ Consistent with this theoretical perspective, the present study therefore focuses on examining these volitional moderators among adolescents receiving mental healthcare.

**Fig. 1:**
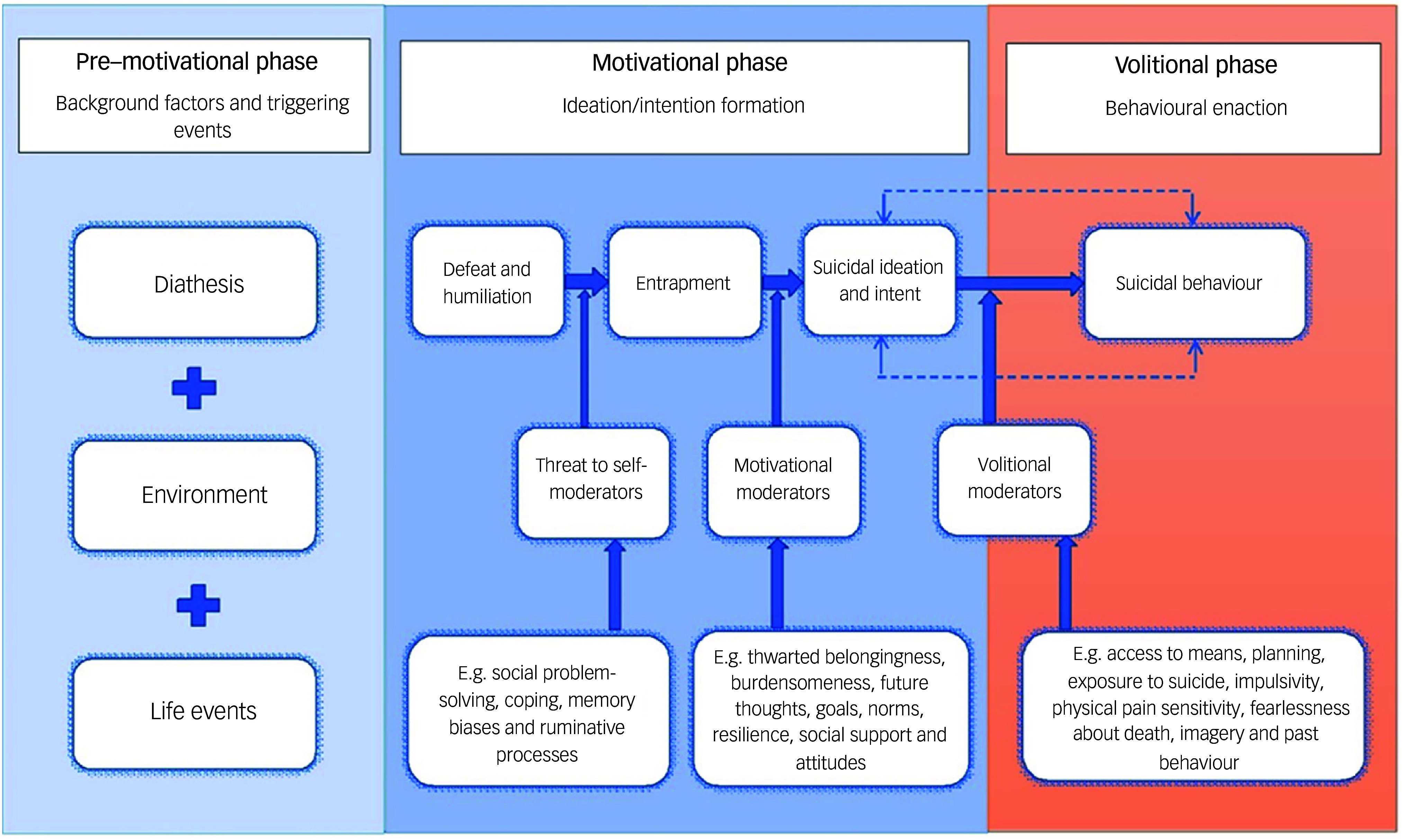
Fig. 1 long description. The integrated motivational–volitional model of suicidal behaviour.^10^

The IMV model consists of three phases: the pre-motivational phase, which describes biological and social background factors; the motivational phase, where feelings of defeat and entrapment are prerequisites for developing suicidal ideation; and the volitional phase, which outlines the factors associated with whether individuals act on their suicidal ideation. Volitional moderators include access to means of suicide, exposure to suicidal behaviour, capability for suicide (fearlessness about death and increased physical pain tolerance), planning, impulsivity, mental imagery and past suicidal behaviour. The relative influence of each of these factors on the transition from ideation to behaviour might not be equal in size and effect, and needs additional exploration. Moreover, few studies have tested the IMV model in adolescents, and findings on volitional moderators are limited and inconsistent, indicating a need for further study.^11^

Volitional moderators have been proposed as key factors in the transition from suicidal ideation to behaviour and may therefore be clinically relevant within psychosocial assessment approaches. Accordingly, the primary aim of this study is to test the core pathways (defeat → entrapment → suicidal ideation → suicide attempts) of the IMV model using path analysis in a population of adolescents receiving mental healthcare. The secondary aim is to examine the extent to which volitional moderators are differentially associated with suicidal ideation and suicide attempts.

## Method

### Participants and procedure

In this cross-sectional study, adolescents (aged 13–19 years) were recruited from the Child and Adolescent Psychiatry Department in Odense, Denmark, between May 2023 and May 2024. Adolescents were eligible if they were either newly referred to the department or currently receiving psychiatric care. All eligible patients were invited to complete a battery of questionnaires (see Measures, below). The range of diagnoses among participants (*N* = 203) was broad, including depression, anxiety, obsessive–compulsive disorder, developmental disorders and eating disorders. Patients were informed about the study via email sent prior to their consultation. They had the option of completing the electronic survey (SurveyXact) either before or after their visit to the department. If patients chose to complete the questionnaire at the department, they had access to healthcare personnel for support, if required. Due to poor health, dyslexia, time constraints or other issues, 20 participants (9%) did not complete the questionnaires and were excluded. All participants were informed about the study’s aim, written informed consent was obtained from all participants and all data were fully anonymised.

### Ethical standards

The authors assert that all procedures contributing to this work comply with the ethical standards of the relevant national and institutional committees on human experimentation, and with the Helsinki Declaration of 1975 as revised in 2013. Child-written consent was collected, because the study was conducted under GDPR Article 6(1)(e) and Section 10 of the Danish Data Protection Act, which allow scientific research to be conducted without relying on parental consent. Parents were informed about the study. The study was approved by the Research & Innovation Organisation at the University of Southern Denmark under journal number 11.809.

### Measures

The battery of questionnaires included a range of validated (apart from exposure to suicidal behaviour and mental imagery) psychometric scales that were translated into Danish following best-practice translation guidelines, specifically the forward/backward translation procedure.^12^ The questionnaires were pilot-tested among younger students (aged 13–15 years) to assess their suitability and identify any potential errors.

The patients were asked about their age and gender (male, female, other), and the following scales were used.

#### Scales used to assess the core pathways of the IMV model

Defeat and entrapment. Defeat was assessed using four items from the Short Defeat and Entrapment Scale.^13^ Responses are rated on a 5-point Likert-type scale, ranging from 0 (not at all like me (completely disagree)) to 4 (extremely like me (completely agree)), and are answered according to how the participant has felt over the past 2 months. The four-item Entrapment Scale–Short Form^14^ was used to measure entrapment, with the same response options as for defeat and entrapment.

Suicidal ideation and behaviour. The Self-Injurious Thoughts and Behaviours Interview Scale^15^ was used to assess the presence, frequency and characteristics of a participant’s history of suicidal thoughts, non-suicidal-self-injury (NSSI) and suicide attempts. Participants completed the following questions: ‘Have you ever seriously considered taking your own life?’ (suicidal ideation), ‘Have you ever harmed yourself without any intention of dying?’ (NSSI) and ‘Have you ever attempted to kill yourself where you at least had some wish of dying?’ (suicide attempt). Response options ranged from ‘never’ to ‘five or more times’. For suicidal ideation and suicide attempts, responses were dichotomised into absence (never) versus presence (one or more occurrences). For NSSI, a dichotomous variable reflecting repeated NSSI was created, defined as more than one episode (two or more occurrences), in order to capture more persistent self-injurious behaviour.

Exposure to suicidal behaviour. Respondents were asked two questions about their experiences with family and/or close friends engaging in suicidal behaviour: ‘Has anyone among your family attempted suicide, deliberately harmed or killed themselves?’; and ‘Has anyone among your close friends attempted suicide or deliberately harmed or killed themselves?’ The answers to both items ranged from 0 to 2: 0, no; 1, yes, within the past 12 months; and 2, yes, more than 12 months ago.^16^ We used a dichotomised version of exposure to suicidal behaviour (unexposed (0), ever exposed (1)).

Impulsivity. An abridged version of The Barrett Impulsiveness Scale (BIS-11) was used to measure impulsivity.^17,18^ We used 5 items to measure motor impulsiveness because this reflects behavioural disinhibition (e.g. ‘I do things without thinking’), and responses were made on a 4-point scale ranging from 1, rarely/never, to 4, almost always/always.

Pain tolerance and fearlessness about death. The Acquired Capability with Rehearsal for Suicide Scale was used to measure pain tolerance and fearlessness about death.^19^ This scale has 7 items and was rated on a 9-point scale ranging from 0, not at all (completely disagree) to 8, very strongly (completely agree).

Mental imagery. Four items measuring mental images about one’s own death were drawn from Holmes et al.^20^ These items assess mental images of past self-harm, imagined future self-harm and the consequences of one’s death. The items were rated on a Likert-type scale from 0, never to 5, always.

Multi-item constructs (e.g. volitional moderators, defeat and entrapment) were derived from validated scales, with item responses summed to generate total scores in accordance with standard scoring procedures. These total scores were treated as continuous variables and were included in both the path analysis and multinomial logistic regression analyses. Variables that were inherently dichotomous (e.g. suicidal ideation, suicide attempts and exposure to suicidal behaviour measures) were included as such. No dichotomisation of continuous variables was applied in the main analyses.

In addition, dichotomous versions of each volitional moderator were created to examine cumulative effects. Exposure to suicidal behaviour and NSSI were included as dichotomous variables as defined by the original measures (i.e. presence versus absence, with NSSI reflecting repeated behaviour). For the remaining volitional moderators, dichotomisation was based on median cut-off values derived from a separate study conducted in a non-clinical adolescent population.^21^ The thresholds were: impulsivity (>11), pain tolerance (>8), fearlessness about death (>22) and mental imagery (>8). These cut-offs were used to define relatively higher versus lower levels of each construct, under the assumption that they better reflect normative variation than cut-offs derived from the present clinical sample.

Based on the dichotomised version of the volitional moderators, a cumulative dose–response variable was created by summing the number of dichotomised volitional moderators present, resulting in a score ranging from 0 to 6. This variable was subsequently included as a predictor in a separate multinomial logistic regression analysis to examine whether increasing exposure to volitional moderators was associated with suicidal ideation and suicide attempts.

### Statistical analysis

We conducted *a priori* power calculations using effect size estimates from a related IMV-based adolescent suicide risk study.^22^ These indicated that a sample of 200 participants was sufficient to test the main IMV pathways and detect a dose–response pattern across volitional moderators. Descriptive statistics, including minimum, maximum, means and standard deviations, were calculated for all study variables. Pearson’s and Spearman’s correlation coefficients were computed to assess the associations between all study variables (see Supplementary Table 1 available at https://doi.org/10.1192/bjo.2026.12066). The internal consistency of scales used in the study was evaluated using Cronbach’s alpha coefficient, with a value of ≥0.70 considered acceptable.

Path analysis was conducted to examine associations between variables in the core pathways of the IMV model (defeat → entrapment → suicidal ideation → suicide attempts). This approach allows for the estimation of both direct and indirect effects between variables, thereby testing the hypothesised processes underlying the transition from motivational to volitional phases. Gender and age were included as confounders. Gender was included as a categorical variable using dummy coding with females as the reference group, resulting in separate coefficients for males and individuals identifying as ‘other’. The initial model was informed by the IMV framework and included all theoretically plausible forward pathways between variables. The core pathways of the IMV model were retained in all models. The optimal model, which included significant pathways and the best-fitting statistics, was then selected using the criteria recommended by Rosseel.^23^ Standardised coefficients are reported to allow comparison of effect sizes across pathways. The lavaan package (version 0.7-2, Windows 11; Yves Rosseel and the lavaan project, Ghent, Belgium; https://cran.r-project.org/package=lavaan) in R (version 4.4.3, Windows 11; R Foundation for Statistical Computing, Vienna, Austria; https://www.r-project.org) was used to estimate pathway effects and calculate model fit statistics. The Broyden–Fletcher–Goldfarb–Shannon optimisation method with diagonally weighted least squares estimation was applied to obtain robust standard errors.^23^

We employed multinomial logistic regression modelling to analyse crude and adjusted associations between volitional moderators (the predictors) and the three mutually exclusive outcomes: no suicidal ideation, suicidal ideation only and suicide attempts (irrespective of ideation). No suicidal ideation (i.e. no history of suicidal ideation or suicide attempts) was set as the reference category. The model provided odds ratios, confidence intervals, *p*-values and probability with confidence intervals for the outcomes. Odds ratios are reported in tables, and probability visualised in figures, showing the probability of the outcome as a function of the level of the volitional moderator. For formal assessment of differences in associations between suicidal ideation and suicide attempts, Wald tests were conducted to compare the corresponding regression coefficients across outcome groups; the mlogistic command in Stata (Stata/BE version 18, Windows 11; StataCorp LLC, College Station, Texas, USA; https://www.stata.com) was used for this analysis.

### Volitional moderator (dose–response version)

Consistent with the IMV model’s emphasis on multiple volitional moderators jointly facilitating enactment, we created a dose–response variable (count of dichotomised volitional moderators) and estimated multinomial logistic models across the three outcomes (no suicidal behaviour, suicidal ideation only, suicide attempts). This approach mitigates multicollinearity among correlated moderators, aligns with the model’s cumulative risk premise and yields clinically interpretable gradients of risk. We report odds ratios and predicted probabilities. This cumulative dose–response operationalisation follows established methods in the cumulative risk literature.^24,25^ We did not stratify or adjust for psychiatric diagnoses, to allow evaluation of the IMV model’s functioning in a broad clinical adolescent psychiatric population.

## Results

A total of 223 participants took part in the study. Participants who did not complete the survey (20 (9.0%)) were excluded from the analysis. The final sample consisted of 203 participants (72.9% female, 21.2% male and 5.9% other gender). Participants’ ages ranged from 13 to 19 years (mean 16.40, s.d. 1.70).

Of the 203 participants, 141 (69.5%) reported having serious thoughts about killing themselves, 135 (66.5%) reported NSSI and 68 (33.5%) reported having made a suicide attempt at some stage in in their lifetime. Sixteen (7.9%) had engaged in NSSI and 27 (13.3%) had attempted suicide only once in their lives; 119 (58.6%) engaged in repeated NSSI and 41 (20.2%) had made multiple suicide attempts. Sixty-seven (47.5%) of those with suicidal ideation had attempted suicide; 61 (30.05%) reported no history of suicidal ideation or suicide attempts (see Supplementary Table 2).

Descriptive statistics and Cronbach’s *α* coefficients for all the scales used in this study are given in Supplementary Table 3. All measures demonstrated acceptable levels of internal consistency, with *α* coefficients ranging from 0.71 to 0.89.

### Testing the core pathways of the IMV model

**Fig. 2:**
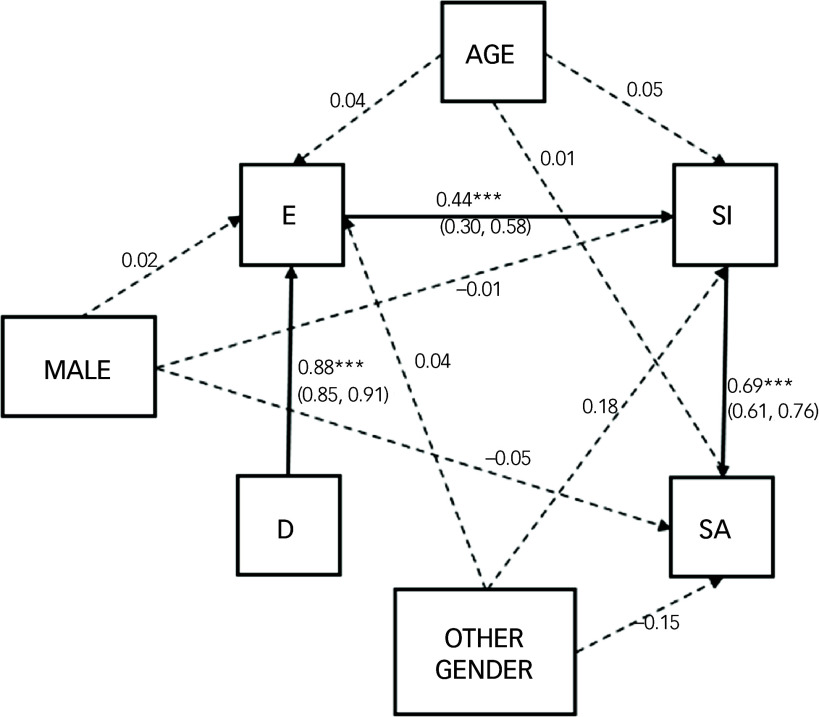
Standardised path coefficients and confidence intervals for the core pathways of the integrated motivational-volitional model, with suicide attempt as the outcome, adjusted for age and gender. E, entrapment; SI, suicidal ideation; D, defeat; SA, suicide attempt. ***p < 0.001.

Figure 2 shows that the standard path coefficients have a strong, significant relationship with lifetime suicide attempts, ranging from 0.44 to 0.88 (*p* < 0.001). Among the paths (defeat → entrapment → suicidal ideation → suicide attempts), the strongest was from defeat to entrapment (0.88, *p* < 0.001) and the weakest was from entrapment to suicide ideation (0.44, *p* < 0.001). Neither of the confounders (age, male and other gender) was significant. Male gender had a negative path coefficient for suicide ideation (−0.01) and suicide attempts (−0.05), and other gender had a negative path coefficient on suicide attempts (−0.15). The model statistics were Comparative Fit Index 1.00, Tucker–Lewis Index 1.02, root mean square error of approximation 0, standardised root mean square residual 0.02 and Normed Fit Index 1.02, indicating a very good fit.

### Associations between volitional moderators and suicidal ideation and suicide attempts

As shown in Table 1, almost all volitional moderators were significantly associated with both suicidal ideation and suicide attempts in the crude analyses, with the exception of impulsivity, which was not significantly associated with suicidal ideation. Odds ratios cannot be directly compared across different volitional moderators due to differences in scaling; however, they can be compared across outcomes because they share a common reference category (no suicidal behaviour), and these differences were formally investigated using Wald tests.

**Table 1:** Crude and adjusted odds ratios, confidence intervals and *p*-values from multinominal logistic regression, with no suicidal behaviour as the reference category Table 1 long description.

Variables	Crude odds ratio (no suicidal behaviour as reference)		Wald test (*p*-value)	Adjusted odds ratio (no suicidal behaviour as reference)		Wald test (*p*-value)
	Suicidal ideation only	Suicide attempt		Suicidal ideation only	Suicide attempt	
Factors	*n* = 74 (36.45%)	*n* = 68 (33.50%)		*n* = 74 (36.45%)	*n* = 68 (33.50%)	
Confounders						
Age	1.05 (0.86–1.29)	1.11 (0.90–1.36)	0.591	1.14 (0.87–1.49)	1.11 (0.82–1.52)	0.836
Male (versus female)	0.73 (0.33–1.63)	0.53 (0.22–1.24)	0.457	1.04 (0.33–3.24)	0.95 (0.23–3.87)	0.863
Other gender (versus female)	5.79 (0.69–48.9)	3.25 (0.35–30.1)	0.378	9.45 (0.88–101.4)	4.72 (0.30–74.4)	0.373
Volitional moderators						
Exposure to suicidal behaviour	2.61* (1.29–5.29)	8.28** (3.39–20.20)	**0.011**	0.97 (0.37–2.53)	1.29 (0.36–4.60)	0.593
Impulsivity	1.05 (0.95–1.16)	1.17* (1.06–1.29)	**0.017**	0.98 (0.87–1.11)	1.05 (0.91–1.21)	0.182
Pain tolerance	1.11* (1.01–1.22)	1.26** (1.13–1.40)	**0.003**	1.02 (0.90–1.15)	1.03 (0.89–1.19)	0.858
Fearlessness about death	1.20** (1.13–1.26)	1.30** (1.21–1.40)	**<0.0001**	1.17** (1.08–1.26)	1.23** (1.13–1.34)	**0.049**
Mental imagery	1.33** (1.17–1.49)	1.57** (1.38–1.79)	**<0.0001**	1.15 (0.97–1.35)	1.23* (1.03–1.47)	0.162
Repeated NSSI (ever)	5.42** (2.51–11.7)	38.15** (13.5–108)	**<0.0001**	3.74* (1.46–9.89)	19.67** (5.38–72.0)	**0.003**
Dose–response	2.21** (1.67–2.93)	3.94** (2.74–5.67)	**<0.0001**	–	–	
IMV core factors						
Defeat	1.14* (1.05–1.23)	1.23** (1.13–1.34)	**0.047**	–	–	
Entrapment	1.16** (1.08–1.25)	1.26** (1.15–1.37)	0.058	–	–	

NSSI, non-suicidal-self-injury; IMV, integrated motivational–volitional model. Odds ratios represent the relative change in odds of the outcome compared with the reference category (no suicidal behaviour) for a one-unit increase in exposure. *p < 0.05, **p < 0.001, Wald test comparison of coefficients between the suicidal ideation and suicide attempt groups and significant differences are marked by bold *p*-value. For the dose–response variable, the odds ratio represents the relative change in odds associated with each one-unit increase (i.e. one additional volitional moderator).

Based on crude estimates, volitional moderators were consistently more strongly associated with suicide attempts than with suicidal ideation, as reflected by higher odds ratios for the former across all moderators. Wald tests indicated that these differences between outcomes were statistically significant. Repeated NSSI showed particularly strong associations, with substantially higher odds ratios for suicide attempts than for suicidal ideation (odds ratio 38.15 *v*. 5.44).

**Fig. 3:**
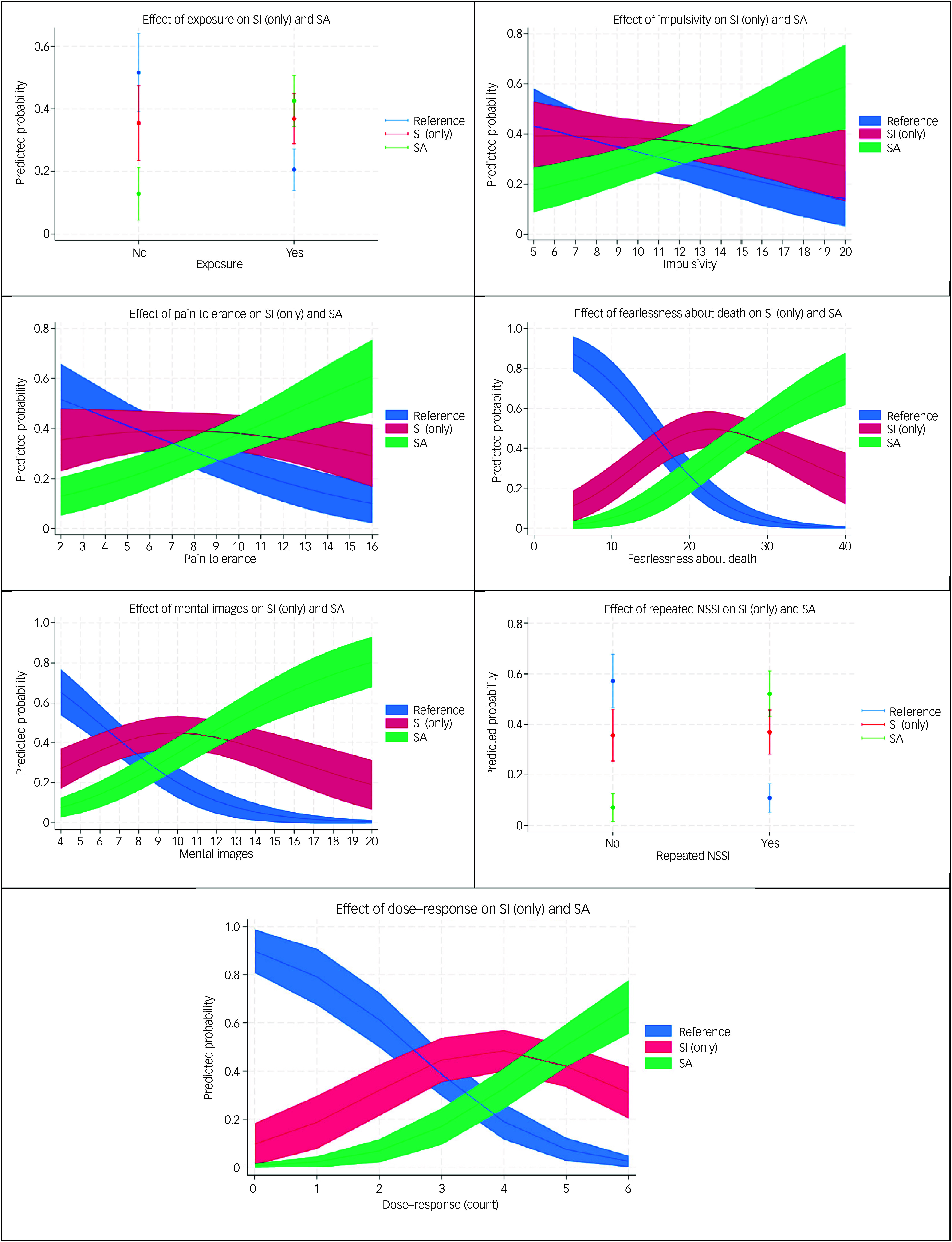
Fig. 3 long description. Probability of each outcome (no suicidal behaviour, suicide ideation only and suicide attempts), including confidence intervals, as a function of levels of exposed volitional moderators, for the six analysed volitional moderators (exposure to suicidal behaviour, impulsivity, pain tolerance, fearlessness about death, mental images and repeated non-suicidal-self-injury (NSSI)), and for the number of exposed volitional moderators (dose–response). SI, suicidal ideation; SA, suicide attempt.

Figure 3 further illustrates this pattern, showing that the likelihood of a suicide attempt is minimal when volitional moderators are at low levels, whereas the likelihood of experiencing suicidal ideation is slightly higher under the same conditions. Conversely, the probability of a suicide attempt increases significantly with high levels of volitional moderators. This pattern is consistent across all six volitional moderators, showing that the likelihood of suicide attempts and suicidal ideation increases as the level of volitional moderators rises, with the highest increase noted for suicide attempts.

By contrast, in the adjusted analyses, fewer volitional moderators remained significantly associated with outcomes. Specifically, fearlessness about death and repeated NSSI were independently associated with both suicidal ideation and suicide attempts, with stronger associations observed for the latter, as confirmed by Wald tests.

Finally, higher levels of defeat and entrapment were associated with both outcomes; however, only defeat showed a significantly stronger association with suicide attempts.

### Dose–response volitional moderator

The dose–response volitional moderator (number of volitional moderators to which an individual was exposed (0–6)) was associated with suicidal ideation and suicide attempts, and significantly more strongly for the latter (average odds ratio 3.94 [CI 95%: 2.74–5.67, *p* < 0.001]) than for the former (odds ratio 2.21 [CI 95%: 1.67–2.93, *p* < 0.001]) per one unit change in the number of volitional moderators (Table 1). The association between the dose–response volitional moderator and probability for the outcome is visualised in Fig. 3. Individuals exposed to a low number of volitional moderators (0–1) are more likely to have no suicidal behaviour as outcome, compared with those exposed to more than one. Individuals exposed to a moderate number of volitional moderators (2–4) are most likely to have suicide ideation as outcome, and those exposed to the highest number of volitional moderators (5–6) are most likely to have suicide attempt as the outcome.

## Discussion

Our findings provide support for the core pathways of the IMV model of suicidal behaviour among adolescents in clinical psychiatric care. Although the study design was cross-sectional, the findings are consistent with the model’s core pathways – which posit that defeat leads to entrapment, subsequently leading to suicidal ideation and ultimately to suicide attempts. The strongest pathway identified was from defeat to entrapment, with that from entrapment to suicidal ideation being the weakest. Volitional moderators, including exposure to suicidal behaviour, impulsivity, fearlessness about death, mental imagery of one’s own death, pain tolerance and repeated NSSI, were associated with both suicidal ideation and suicide attempts, with significantly stronger associations observed for the latter. A dose–response analysis of these volitional moderators revealed that high cumulative exposure was significantly associated with increased risk of suicide attempts compared with suicidal ideation.

### Core pathways of the IMV model

As highlighted in a recent systematic review, although a growing number of studies have tested and supported the core pathways of the IMV model, there have been few tests conducted within a clinical psychiatric adolescent population.^11^ Our examination of the IMV model among adolescents yielded support for it, although the pathway from entrapment to suicidal ideation was the weakest. Our findings cannot be explained by demographic differences, and are different from those in a study of adolescents in China where the defeat-to-entrapment pathway was the weakest (path coefficient 0.13, *p* < 0.001).^22^ Conversely, a large-scale study of university students in the UK found that the pathways from entrapment to suicidal ideation (standardised regression weight 0.20, *p* < 0.001), and from suicidal ideation to suicide attempts (standardised regression weight 0.30, *p* < 0.001), exhibited weak to moderate positive relationships.^21,26^ However, in both studies, the associations were statistically significant. A Danish study showed similar results.^21^

Other ideation-to-action models propose alternative pathways to suicidal ideation. The interpersonal theory of suicide^27,28^ posits that the combination of thwarted belongingness and perceived burdensomeness leads to suicidal desire. Similarly, the three-step theory suggests that the combination of psychological pain and hopelessness leads to suicidal ideation.^8^ We briefly reference other ideation-to-action models because these highlight alternative, theoretically established routes to suicidal ideation driven by other factors that may overlap conceptually with defeat and entrapment.

### Volitional moderators

Our findings suggest that the volitional moderators are more strongly associated with suicide attempts than with suicidal ideation, consistent with the IMV model. In all of the probability plots, higher levels of volitional moderators corresponded to increased probabilities of suicide attempts compared with suicidal ideation. Despite extensive research efforts to predict suicidal behaviour based on risk factors, the outcomes have been suboptimal.^2^ To contextualise the value of risk factors appropriately, it is essential that we continue to categorise these as being associated with either suicidal ideation or suicidal behaviour. Shifting the focus from risk factors to understanding behavioural enaction specifically could have significant clinical implications, with volitional moderators being part of the broader category of factors related to suicide capability.^29,30^ Suicide capability is generally understood to encompass three main components: acquired capability (reduced fear and increased pain tolerance), dispositional factors (greater susceptibility to suicidal behaviour) and practical factors (ease of acting on suicidal thoughts).^30^ The extensive list of factors related to suicide capability warrants further exploration. The present study contributes to this body of work within the context of the IMV model, highlighting the utility of volitional moderators and emphasising their potential for enhanced distinguishing between those who think about suicide and those who act on their thoughts of suicide.^11^

### Dose–response volitional moderator factor

This study represents the first investigation of a dose–response operationalisation of volitional moderators within the IMV model. The findings suggest a clear and cumulative association between a high number of volitional moderators and the occurrence of suicide attempts, while simultaneously showing a low risk for suicidal ideation on its own. The dose–response volitional moderator factor may hold clinical value for potential inclusion in suicide psychosocial assessments; however, further research is necessary because these findings are preliminary. In addition, agreeing the thresholds for dichotomising the factors requires more detailed exploration, and a weighted dose–response version, where each moderator is not equally weighted, might be preferable. Interaction and non-linear effects also warrant further exploration to avoid oversimplifying what is inherently a complex psychological system. In this current study, a sensitivity analysis of the dose–response volitional moderator factor, excluding repeated NSSI, did not alter the overall pattern and significance.

### Clinical implications

Utilising the IMV model to structure psychosocial assessments for suicidal behaviour could enhance clinicians’ understanding of the relevance of patients’ risk factors in the suicidal process, and aid in distinguishing between individuals with suicidal ideation and those at higher risk of attempting suicide. Indeed, this would be consistent with recent calls to move towards more therapeutic assessment and clinical formulation approaches.^4^ To this end, there have been efforts to develop an IMV-informed clinical formulation tool that emphasises mapping out the risk factors according to their phase-specific roles (i.e. ideation versus enactment).

Assessing volitional moderators is likely to be crucial, because these appear to be more strongly associated with suicide attempts than suicidal ideation and might therefore help identify individuals at higher risk for suicide. Although incorporating volitional moderators into psychosocial assessments may not fully address the challenge of low positive predictive value in risk assessment tools, it may enhance the quality of the assessments.^31^ Clinically, the number of co-occurring volitional moderators may be more useful to consider than any single moderator, although repeated NSSI, fearlessness about death and death-related imagery in this study provided the clearest independent signals.

Finally, because the effects of some of the volitional moderators, such as suicide-related mental imagery and social modelling, are potentially modifiable, they could be targeted in treatment. Such an approach aligns with taking a dynamic and patient-centred approach to suicide risk assessment.^10,32^

### Limitations

Although this study has many strengths (e.g. focus on an adolescent psychiatric sample and in-depth exploration of the IMV model constructs), its cross-sectional nature precluded investigation of causality or temporal sequences. Additionally, some of the factors, such as fearlessness about death or pain tolerance, are dynamic, varying across both time and context. As a result, the findings represent only a snapshot in time.^33,34^ In addition, some of the key variables were lifetime measures, thereby limiting the model fit for time-specific occurrences.

The study had sufficient power to calculate statistically significant estimates of standard path coefficients (with narrow confidence intervals) for the core pathways of the IMV model, and to detect the strong dose–response pattern of volitional moderators. Indeed, the count-based dose–response index demonstrated a clear, monotonic increase in probability for suicide attempt. Although statistically significant differences between suicidal ideation and suicide attempts were observed for several volitional moderators in the crude analyses, these differences were less consistent in the adjusted models. This probably reflects reduced power to detect differences between outcome groups when multiple correlated variables are included simultaneously in multinomial models, as well as attenuation of individual effect estimates due to shared variance between volitional moderators and the inclusion of strong correlates such as repeated NSSI. We did not include planning and access to means, because we chose to focus on the more psychosocial moderators.

The study population was limited to adolescents in contact with a single psychiatric department in Denmark. It was also heterogeneous, including individuals with a wide range of mental health diagnoses as well as those who had yet to be assessed and those with low levels of psychiatric symptoms.

### Findings

In a clinical adolescent sample, this cross-sectional study highlights the utility of the IMV model within the suicidal process. The findings also point to the potential of IMV volitional moderators in distinguishing between those with suicidal ideation and those who have attempted suicide. Specifically, we observed a dose–response pattern: as exposure to the number of volitional moderators increased so did the likelihood of attempted suicide. These findings are preliminary and should be replicated in large, multi-wave, longitudinal and ecological momentary assessment studies.

## Supporting information

10.1192/bjo.2026.12066.sm001 Christiansen et al. supplementary material 1 Christiansen et al. supplementary material

10.1192/bjo.2026.12066.sm002 Christiansen et al. supplementary material 2 Christiansen et al. supplementary material

10.1192/bjo.2026.12066.sm003 Christiansen et al. supplementary material 3 Christiansen et al. supplementary material

## Data Availability

The data that support the findings of this study are not publicly available due to the lack of participant consent for third-party data transfer. The data are therefore not available upon request. Researchers interested in further analyses are welcome to contact the corresponding author (E.C.) to discuss potential scientific collaboration, with any additional analyses conducted by the original research team in accordance with ethical and legal restrictions.

## Supplementary material

The supplementary material is available online at https://doi.org/10.1192/bjo.2026.12066

## Fig. 1 Long description

The flowchart illustrates the stages of suicidal behavior according to the integrated motivational-volitional model. It is divided into three main phases: Pre-motivational phase, Motivational phase, and Volitional phase. The Pre-motivational phase includes Diathesis, Environment, and Life events. The Motivational phase includes Defeat and humiliation, Entrapment, Threat to self-moderators, Motivational moderators, and Suicidal ideation and intent. The Volitional phase includes Volitional moderators and Suicidal behavior. Defeat and humiliation lead to Entrapment, which then leads to Suicidal ideation and intent. Threat to self-moderators and Motivational moderators influence Entrapment and Suicidal ideation and intent. Suicidal ideation and intent lead to Suicidal behavior, which is influenced by Volitional moderators.

Navigate back to Fig. 1.

## Table 1 Long description

A table comparing crude and adjusted odds ratios for suicidal ideation and suicide attempts, with no suicidal behavior as the reference category. The table has 12 rows and 10 columns. Column headers are: Variables, Crude odds ratio (Suicidal ideation only), Crude odds ratio (Suicide attempt), Wald test (p-value), Adjusted odds ratio (Suicidal ideation only), Adjusted odds ratio (Suicide attempt), and Wald test (p-value). Row labels include Factors, Confounders, Volitional moderators, and MV core factors. Each row provides specific values for the odds ratios and p-values. Notable trends include significant associations for most volitional moderators with both suicidal ideation and attempts, except for impulsivity, which is not significantly associated with suicidal ideation.

Navigate back to Table 1.

## Fig. 3 Long description

Panel A: A scatter plot shows the effect of exposure on suicidal ideation (SI) only and suicide attempts (SA). The x-axis is labeled Exposure with categories No and Yes, and the y-axis is labeled Predicted probability. Three data series are shown: Reference, SI (only), and SA. Panel B: A line graph illustrates the effect of impulsivity on SI only and SA. The x-axis is labeled Impulsivity with a range from 5 to 20, and the y-axis is labeled Predicted probability. Three lines represent Reference, SI (only), and SA, with shaded areas indicating confidence intervals. Panel C: A line graph depicts the effect of pain tolerance on SI only and SA. The x-axis is labeled Pain tolerance with a range from 2 to 16, and the y-axis is labeled Predicted probability. Three lines represent Reference, SI (only), and SA, with shaded areas indicating confidence intervals. Panel D: A line graph shows the effect of fearlessness about death on SI only and SA. The x-axis is labeled Fearlessness about death with a range from 10 to 40, and the y-axis is labeled Predicted probability. Three lines represent Reference, SI (only), and SA, with shaded areas indicating confidence intervals. Panel E: A line graph illustrates the effect of mental images on SI only and SA. The x-axis is labeled Mental images with a range from 4 to 20, and the y-axis is labeled Predicted probability. Three lines represent Reference, SI (only), and SA, with shaded areas indicating confidence intervals. Panel F: A scatter plot shows the effect of repeated non-suicidal self-injury (NSSI) on SI only and SA. The x-axis is labeled Repeated NSSI with categories No and Yes, and the y-axis is labeled Predicted probability. Three data series are shown: Reference, SI (only), and SA. Panel G: A line graph depicts the effect of dose-response on SI only and SA. The x-axis is labeled Dose-response with a range from 0 to 6, and the y-axis is labeled Predicted probability. Three lines represent Reference, SI (only), and SA, with shaded areas indicating confidence intervals.

Navigate back to Fig. 3.
