# Supplementary material for: Testing the integrated motivational–volitional model of suicidal behaviour among Danish adolescents in clinical psychiatric care: focus on volitional moderators
Source: BJPsych Open. 2026 Aug 10;12(5):e209. doi: 10.1192/bjo.2026.12066 (PMC13458380; doi:10.1192/bjo.2026.12066)
Supplement: Supplementary file 1 [file S2056472426120663sup001.docx]

Table S1. Bivariate correlations between all variables included in the study

|  | 1 | 2 | | 3 | | 4 | | 5 | | 6 | | 7 | | 8 | | 9 | | 10 | | 11 | |
| --- | --- | --- | --- | --- | --- | --- | --- | --- | --- | --- | --- | --- | --- | --- | --- | --- | --- | --- | --- | --- | --- |
| 1. Defeat |  | |  | |  | |  | |  | |  | |  | |  | |  | |  | |  |
| 2. Entrapment | .88*** |  | |  | |  | |  | |  | |  | |  | |  | |  | |  | |
| 3. Suicidal ideation | .32*** | .37*** | |  | |  | |  | |  | |  | |  | |  | |  | |  | |
| 4. Suicidal attempt | .26*** | .29*** | | .45*** | |  | |  | |  | |  | |  | |  | |  | |  | |
| 5. Exposure to suicidal behaviour | .17* | .22** | | .30*** | | .29*** | |  | |  | |  | |  | |  | |  | |  | |
| 6. Impulsivity | .20** | .28*** | | .18** | | .24*** | | .25*** | |  | |  | |  | |  | |  | |  | |
| 7. Pain tolerance | .09 | .18** | | .31*** | | .32*** | | .28*** | | .33*** | |  | |  | |  | |  | |  | |
| 8. Mental images | .57*** | .56*** | | .49*** | | .44*** | | .33*** | | .31*** | | .28*** | |  | |  | |  | |  | |
| 9. Fearlessness about death | .46*** | .52*** | | .61*** | | .49*** | | .37*** | | .23* | | .41*** | | .63*** | |  | |  | |  | |
| 10. Repeated NSSI | .24*** | .24*** | | .49*** | | .47*** | | .40*** | | .16* | | .37*** | | .41*** | | .43*** | |  | |  | |
| 11. Age | .05 | .07 | | .05 | | .06 | | .06 | | .09 | | .05 | | .11 | | .09 | | .05 | |  | |

*p<0.05; **p<0.01; ***p<0.001; NSSI: Non-Suicidal-Self-Injury
