# Supplementary material for: Testing the integrated motivational–volitional model of suicidal behaviour among Danish adolescents in clinical psychiatric care: focus on volitional moderators
Source: BJPsych Open. 2026 Aug 10;12(5):e209. doi: 10.1192/bjo.2026.12066 (PMC13458380; doi:10.1192/bjo.2026.12066)
Supplement: Supplementary file 2 [file S2056472426120663sup002.docx]

Table S2. Prevalence of suicidal ideation, NSSI and suicide attempts by gender and timeframe

|  | **Suicidal ideation** |  | **NSSI** |  | **Suicide attempt** |
| --- | --- | --- | --- | --- | --- |
|  | n (%) |  | n (%) |  | n (%) |
| **Lifetime** |  |  |  |  |  |
| Total | 141 (69.5) |  | 135 (66.5) |  | 68 (33.5) |
| Males | 26 (60.5) |  | 21 (48.8) |  | 11 (25.6) |
| Females | 104 (70.3) |  | 104 (70.3) |  | 53 (35.8) |
| Other | 11 (91.7) |  | 10 (83.3) |  | 4 (33.3) |
| Once | - |  | 16 (7.9) |  | 27 (13.3) |
| Multiple times | - |  | 119 (58.6) |  | 41 (20.2) |
| **In the past year** |  |  |  |  |  |
| Total | 122 (60.1) |  | 105 (51.3) |  | 39 (19.2) |
| Males | 19 (44.2) |  | 17 (39.5) |  | 7 (16.3) |
| Females | 94 (63.5) |  | 81 (54.7) |  | 29 (19.6) |
| Other | 9 (75.0) |  | 7 (58.3) |  | 3 (25.0) |
| **In the past month** |  |  |  |  |  |
| Total | 83 (40.9) |  | 58 (28.6) |  | 16 (7.9) |
| Males | 13 (30.2) |  | 8 (18.6) |  | 3 (7.0) |
| Females | 64 (43.2) |  | 48 (32.4) |  | 13 (8.8) |
| Other | 6 (50.0) |  | 2 (16.7) |  | 0 |
