# Supplementary material for: Testing the integrated motivational–volitional model of suicidal behaviour among Danish adolescents in clinical psychiatric care: focus on volitional moderators
Source: BJPsych Open. 2026 Aug 10;12(5):e209. doi: 10.1192/bjo.2026.12066 (PMC13458380; doi:10.1192/bjo.2026.12066)
Supplement: Supplementary file 3 [file S2056472426120663sup003.docx]

Table S3. Cronbach's alpha for psychometric variables including mean values, standard deviation and variable range.

| **Psychometric measure** | **Cronback’s alpha** | **No Suicidal**  **Behaviour**  **Mean (sd)** | **Suicidal Ideation**  **Mean (sd)** | **Suicide Attempts**  **Mean (sd)** | **All**  **Mean (sd)** | **All**  **Min-Max** |
| --- | --- | --- | --- | --- | --- | --- |
| *Core pathways* |  |  |  |  |  |  |
| Defeat | 0.89 | 6.31 (4.34) | 9.50 (4.33) | 10.24 (4.23) | 8.53 (4.55) | 0-16 |
| Entrapment | 0.89 | 6.43 (4.40) | 10.33 (4.47)) | 11.06 (4.47) | 9.16 (4.78) | 0-16 |
| Suicide ideation | - | - | - | 0.99 (0.12) | 0.69 (0.46) | 0-1 |
| Suicide attempt | - | - | 0.48 (0.50) | - | 0.33 (0.47) | 0-1 |
| *Volitional moderators* |  |  |  |  |  |  |
| Exposure to suicidal behaviour | - | 0.48 (0.50) | 0.79 (0.41) | 0.88 (0.32) | 0.69 (0.46) | 0-1 |
| Impulsivity | 0.85 | 10.41 (3.80) | 11.85 (3.92) | 12.69 (3.99) | 11.41 (3.92) | 5-20 |
| Pain tolerance | 0.71 | 6.74 (4.11) | 9.41 (4.06) | 10.46 (3.95) | 8.57 (4.26) | 2-16 |
| Fearlessness about death | 0.79 | 13.52 (5.40) | 26.09 (8.80) | 29.07 (8.67) | 22.23 (9.83) | 5-40 |
| Mental images of own death | 0.87 | 6.44 (2.77) | 11.01 (4.36) | 12.51 (4.55) | 9.62 (4.47) | 4-20 |
| Repeated NSSI | - | 0.21 (0.41) | 11.01 (4.36) | 0.91 (0.29) | 0.59 (0.49) | 0-1 |
| *Dose-response* |  |  |  |  |  |  |
| Dose-response (VM) § | - | 2.15 (1.46) | 4.47 (1.42) | 4.99 (1.13) | 3.77 (1.78) | 0-6 |

Note. § Count of volitional moderators; sd: standard deviation
